# Dens Sapientiæ

**Published:** 1885-04

**Authors:** J. W. Clowes


### DENS SAPIENTIÆ.

BY J. W. CLOWES, D. D. S.

I have the habit, while engaged in my professional work, of talking upon the subject of my own thoughts, or one suggested by my patient, and am thus enabled to attract the mind, in some degree, from unpleasant conditions. This faculty involves not only the ability to talk while I work, but to work while I talk, and time and progress, without detriment, keep equal pace.

"Why do we have wisdom teeth? They are useless things—the sources of constant trouble, and an unmitigated nuisance." That was the question and these the assertions of my patient, and this the reply that followed their utterance:

"Wisdom teeth have a sensible designation and providential design. They were wisely given as compensation and as substitution for losses. If they are useless, it is because of their position, and the supernumerary part they have to play. That they are 'sources of trouble' is a matter beyond their control, and this, when rightly judged, should be their vindication rather than their offence. If they are a 'nuisance,' it is by compulsion and not by choice. Before speaking further of those teeth, I premise that, in rare instances they have but a meagre development, and still more rarely have neither development nor eruption at all. This shortcoming is from lack of material, and is no more to be charged against the wisdom teeth than like failures in the central and lateral incisors. Both these classes of teeth, at times, vary greatly from their normal size and shape. In some mouths I have seen wisdom teeth larger than other molars, and in the case of one lady there were

*eight of them*, well formed and of good dimensions. Wisdom teeth are great travelers; their tendencies are always to the front, and when they move they are sure to better their condition. While it is almost the universal belief that the teeth are what you have declared, the truth still remains that *they are the strongest of all the molars*, and as such deserve our appreciation and care. The testimony on this point is abundant, and may not be ignored nor set aside by unreasoning prejudice or senseless clamor. We prove this strength on one hand by the very constitution and structure of the teeth themselves, and on the other by reasoning from analogy. It requires but little observation to discover that first molars are very weak teeth, having from three to five defects in their enamel, the second molars from two to three, while the wisdom teeth have seldom or never more than one. These imperfections are the open doors through which the causes of decay enter and destroy the dental substance. Thus we see, by a fair comparison of the facilities for attack, that wisdom teeth are five times as strong as first molars, and three times as strong as second ones. In the laboratory of Nature, when she sets about the work of production, the things she is longest in evolving and maturing are the staunchest and most lasting. If we consider the fruits, the bough apple and the summer pear have a rapid growth, are insipid in taste, and their duration and flavor are as the evanescence of a dream. The pippin and winter nelis pear develop slowly and surely through the autumn days, and with extended time their excellence increases as their juices are refined by the alchemy of the ripening sun. Do we desire a ship? We would not wisely go for material to the whitewood, the ailanthus or other fast-growing trees, but to the unbending oak that has breasted, for a hundred years, the sunshine and the storm! First molars are the product of infantile growth. Their inception, advance and fulfillment, are expressions of weakness. Their mission is intermediary and transient, and their retention, after the twelfth year, a danger too serious to tolerate. They are the great mischief makers of the human mouth. Crowding, irregularity and decay, are before them and behind them! They are the pivotal centres of dental unrest. Remove these teeth and room and regularity will ensue. Take them away and the approximal sockets will contain no ill. Banish them, and the second molars, stronger in constituents, maturer by time and systemic force, will assume their vacancies. Get rid of them and the wisdom teeth, healthful and strong, without obstruction, will nobly fulfill their destined purpose. There are men in my profession who are always ready to extract wisdom teeth on sight. They have never had an independent thought regarding these teeth. The test of their fathers is their test, and they are content to abide there. Has Nature belied her record in the wisdom teeth? Are twenty years and the fullness of their strength less capable than the puling six or the inchoate twelve? The *Dens Sapientiæ* of human mouths is a freedom-loving tooth. Unjustly restricted, its struggles for relief are fearful to witness. 'Cabined, cribbed, confined,'—held fast in bondage—its throes for liberty will carry all before it, and mind and matter suffer an eclipse. In this condition it is the greatest source of nervous irritation, and here I verily believe does epilepsy have its fountain head. Like many worthy ones we know in this purpose-crossed world, '*Dens Sapientiæ*' is unfortunate through circumstance, and should not be judged by its surroundings. Give it fair play—give them fair play, and the full sum and measure of their merits would be wonderous to behold."

