# Errata

**Published:** 1885-05

**Authors:** 


### ERRATA.

In the article by Dr. Clowes in the April No., page 191, third line from the bottom, read "pockets" for "sockets," and on page 192, fifth line from the top, read "rut" for "test," and the sense will be more complete.

